# The First of Us: *Ophiocordyceps* use a novel scramblase-binding peptide to manipulate zombie ants

**DOI:** 10.1101/2025.09.09.674826

**Published:** 2025-09-19

**Authors:** William C. Beckerson, Steffen Werner, Maite Goebbels, João J. Ramalho, Andrew J. M. Swafford, Ilia Soroka, Sinah T. Wingert, Mike Boxem, Suzan Ruijtenberg, Greg J. Stephens, Sander van den Heuvel, Charissa de Bekker

**Affiliations:** 1 Molecular Microbiology, Environmental Biology, Department of Biology, Utrecht University; Utrecht, the Netherlands.; 2 Developmental Biology, Biodynamics and Biocomplexity, Department of Biology, Utrecht University; Utrecht, the Netherlands.; 3 Experimental Zoology Group, Wageningen University & Research; Wageningen, the Netherlands.; 4 Biology Department, Middlebury College; Middlebury, Vermont, United States of America; 5 Department of Physics and Astronomy, VU University Amsterdam; Amsterdam, the Netherlands.; 6 Biological Physics Theory Unit, OIST Graduate University; Okinawa, Japan

**Keywords:** Neuroparasitology, Neuroethology, Behavior, Invertebrate, Fungus, Parasite, *C. elegans*

## Abstract

Parasite-adaptive manipulation of behavior is a widespread natural phenomenon. While *Ophiocordyceps* zombie fungi are well-known for hijacking ant behavior to increase their fitness, functionally characterizing the biomolecules involved in behavior manipulation remains difficult in these non-model organisms. To circumvent this problem, we have adopted the powerful genetics toolbox of *Caenorhabditis elegans* to identify molecular targets and neurophysiological effects of candidate *Ophiocordyceps* effectors. With this approach, we discovered a novel cysteine-rich, small secreted fungal peptide that binds to well-conserved calcium-dependent scramblase channels in neurons, particularly those associated with sensory tissues. This binding suppressed nematode motor coordination and dampened ant olfactory systems vital for communication. Our findings are the first to directly connect an *Ophiocordyceps* effector with its extended phenotype, while demonstrating the neuroethological role of Scramblase-1.

## Introduction

Reciprocal coevolutionary pressures experienced between parasites and their hosts can lead to the evolution of intense host specialization and unique infection strategies over time^1^. In some cases, the intimate symbiotic relationship between organisms can link the genome of parasites with phenotypes in its hosts, a phenomenon referred to as an extended phenotype^2–3^. Behavior manipulation is a common example of an extended phenotype found across the tree of life^4^. Using effectors to alter host behavior, parasites can increase their own fitness by augmenting the development and/or transmission of parasitic propagules. Fungal parasites are particularly well known for their behavior manipulation strategies, inspiring pop culture media like *The Last of Us*^5^ and *The Girl with All the Gifts*^6^. The biodiverse species complex *Ophiocordyceps unilateralis*^7–8^ is particularly well characterized in its temporal induction of multiple conspicuous behavioral changes in infected ants. First, the fungus disrupts the social behaviors of its host, reducing their communication with nestmates. Next, the hosts are led to abandon their foraging roles and leave the nest, a behavioral manipulation that helps the parasite avoid detection and destruction by nestmate social immunity responses^9–10^. Infected ants then exhibit summit disease^11^, climbing nearby vegetation to a vantage point that aids in fungal fruiting body development and spore dispersal^12^. Finally, the ant bites down irreversibly to the substrate at this elevated position, ensuring it remains anchored after death when the fungus transitions to its reproductive stage. By stimulating its host to climb to a higher location, the fungus is better positioned to spread its spores more effectively on the wind. While these extended phenotypes may seem uniquely adapted to the life cycle of social ant species, examples of similar behavioral changes (e.g., summit disease) can be found in other parasite-host interactions as well^13–15^. This suggests that many behavioral manipulation strategies have evolved convergently^16^, implying that different parasites may exploit the same core neurobiological pathways in animal hosts. Understanding which pathways are involved and how these pathways are exploited by these parasites is an important step towards understanding unifying neuroethological processes in insects, particularly those with agricultural implications. By exploring behavioral effectors and understanding their impact on conserved insect neurobiology, we open the door to the discovery of new biocontrol agents to help combat destructive pests.

While the high degree of biodiversity and life cycles of behavior-manipulating parasites is well described, molecular studies are often hindered by the lack of bioengineering techniques available in these majoritively non-model organisms. The same is true for many of their hosts and other agricultural pests, which often lose natural behaviors and stress resilience under laboratory conditions^17–18^. While multiomic tools can help identify putative behavioral effectors in some of these parasite-host interactions, characterizing their effects *in vivo* remains difficult. To address this problem, we sought to find a workaround by integrating tools from more genetically tractable model organisms like *Caenorhabditis elegans*. This involved the utilization of a Yeast Two-Hybrid (Y2H) system and several behavioral assays involving *C. elegans*. Using these tools, we were able to reverse engineer the function of a putative zombie-making peptide to inform further studies in the native host. Ultimately, we identified a cysteine-rich, small secreted peptide that binds to calcium-dependent scramblase proteins (SCRM-1 and SCRM-2) in the neurological tissues of nematodes. Cys-rich peptides are well-known contributors to the neurotoxic effects of arachnid venoms^19^, and their capacity to interfere with ion channels^20^ could make them efficient insecticidal candidates^21^.

While scramblase lipid channels have traditionally been implicated in membrane remodeling through phospholipid translocation^22^, more recent findings have challenged this limited scope, proposing other roles when expressed in neurons^23–24^. In both mouse and fly models, scramblases were found to be involved in synaptic transmission, with homologs for scramblases 1 and 2 playing a vital role in neurotransmitter vesicle release and accumulation at neuromuscular junctions^23–24^. Moreover, these scramblases were prominently found in fly antenna^24^, indicating a putative association with invertebrate olfactory sensing. Olfactory disruption during infection could facilitate *Ophiocordyceps*-adaptive abandonment of nestmates and social roles by interfering with the host’s ability to detect odors used by ants to communicate^25^. We therefore hypothesized that this scramblase-binding *Ophiocordyceps* peptide is involved in the nest abandonment behavior of manipulated ants. Through a combination of computational modeling, colocalization imaging, and behavioral experiments, we strengthened our Y2H findings by showing that exposure to this peptide causes olfactory sensing-related behavioral changes in *Camponotus floridanus*. By incorporating the robust molecular toolboxes of model organisms like *C. elegans* into our analysis pipeline, we have demonstrated the power of interdisciplinary research and provide an alternative way to study the neuroethology of behavioral effectors in difficult to work with systems. We have also successfully characterized the first *bona fide Ophiocordyceps* behavioral effector.

## Results

### Identification of a putative Ophiocordyceps effector involved in behavioral manipulation

**Fig. 1.**
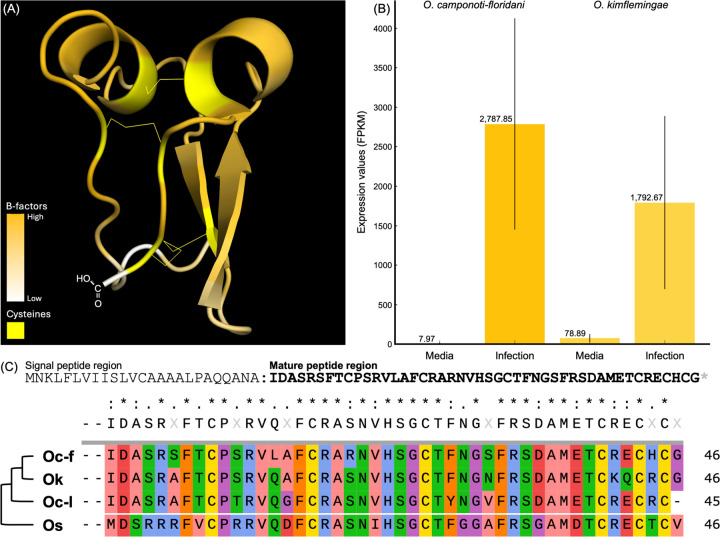
Features of peptide Ophcf2|06345. (A) 3D structural model of Ophcf2|06345 as predicted by AlphaFold2 with high confidence folding shown in orange and low confidence regions shown in white. The position of the cystine residues connected by disulfide bonds are shown in yellow. (B) Expression levels of the Ophcf2|06345 gene in fungi grown in Grace’s insect media compared to infection of carpenter ant hosts. The expression levels for *O. camponoti-floridani* are shown on the left and the expression levels for the homolog in *O. kimflemingae* are shown on the right. (C) Full polypeptide sequence for Ophcf2|06345, top, and the amino acid sequence comparison of the mature peptide regions for homologs found in the *Ophiocordyceps* genus (Oc-f = *O. camponoti-floridani*, Ok = *O. kimflemingae*, Oc-l = *O. camponoti-leonardi*, Os = *O. sinensis*).

It has long been hypothesized that *O. camponoti-floridani* manipulates ant behavior using secreted proteins^9^. We therefore compared the secretome of *O. camponoti-floridani*, determined using a rigorous computational analysis^26^ (Data S1), with transcriptomics data to reveal a novel small secreted peptide (Fig. 1A) putatively involved in *Ophiocordyceps* manipulation of ant behavior. This gene (*Ophcf2|06345* in *O. camponoti-floridani*) showed one of the highest degrees of increased expression during manipulated summiting of *C. floridanus* ants when compared to fungal growth in insect medium (log2-fold change = 8.45^9^; Fig. 1B). Its homolog in *O. kimflemingiae* (Ophio5|1675) also shows a similar expression pattern during *Camponotus castaneus* manipulation (log2-fold change = 4.51^27^; Fig. 1B). Moreover, homologs with predicted secretion signals also exist in other behavior manipulating *Ophiocordyceps* species (Fig. 1C), some of which infect other species of insects (e.g., *Ophiocordyceps sinensis* infection of caterpillars^28^). These homologs all share a high degree of sequence conservation in the mature peptide but show variation in the signal peptide regions. Despite this variation, all homologs are predicted to be secreted (SignalP-6.0; Data S2), suggesting that this putative effector might play an important role in general behavior manipulation. Structural predictions of the secreted peptide from *O. camponoti-floridani* with AlphaFold2 (fig. S1) showed configurational parallels with neurotoxic cys-rich peptides^20^, possessing two ShK-like α-helices^29^ and stable antiparallel β-strands that form a disulfide-directed β-hairpin fold^30^. These equidistant cysteines create three tightly connected disulfide bonds characteristic of stable cysteine knots^19–20^ (Fig. 1A). Furthermore, all of the homologs demonstrated strong conservation of these cysteine residues, which accounted for approximately 13% of the mature peptides, with identical spacing maintained across each of the sequences (Fig. 1C). These features provided promising support for further investigation into potential neurological binding partners in the host.

### Identification of scramblase lipid channels as a target for binding by the putative Ophiocordyceps effector

**Fig. 2.**
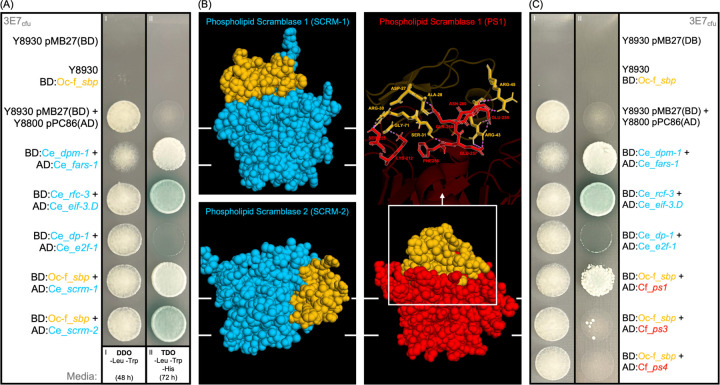
Yeast Two-Hybrid (Y2H) mating assay and computationally predicted binding sites. (A) Mating between yeast strains harboring the coding region for *Ophcf2|06345* protein (SBP) and those harboring the cDNA or ORFeome library from *C. elegans*. The spot assay on the left is plated on double dropout media (DDO) lacking the essential amino acids Leu and Trp to select for successfully mated colonies. The spot assay on the right is plated on triple dropout media (TDO) additionally lacking the amino acid His and with added 3AT to select for robust protein-protein interactions and x-α-Gal to induce a blue colorimetric phenotype. Yeast strains and plasmid names are shown in black text and abbreviated with BD for the binding domain or AD for the activation domain components of the corresponding vectors. *C. elegans* genes are shown in blue, *O. camponoti-floridani* genes in orange, and *C. floridanus* genes in red. Rows 1–2 demonstrate the inability of haploid yeast cells containing the bait vector (pMB27) or the prey vector (pMB27) to grow on either media types. Row 3 demonstrates the ability for diploid mated cells without robust protein-protein interactions to grow on the DDO media, but not the TDO media. Rows 4–6 represent positive controls for the *C. elegans* Y2H libraries, with *dpm-1* x *fars-1* showing weak x-α-gal catabolism, *rfc-3* x *eif-3.D* showing efficient x-α-gal catabolism, and *dp-1* x *ef2–1* showing a very strong rate of x-α-gal catabolism associated with accumulation that affects colony growth. (B) ScanNet predicted binding residues for SBP (orange) with the *C. elegans* scramblases 1 and 2 (blue) and *C. floridanus* scramblase-1 (red). Interacting residues are shown via text overlay alongside their numerical position in each protein. (C) Pairwise mating assay between yeast strains harboring SBP and the *C. floridanus* Phospholipid Scramblases 1, 3, and 4.

To identify candidate binding partners for the Ophcf2|06345 peptide, we performed a series of Y2H assays with cDNA and ORFeome libraries prepared from the *C. elegans* genome. These screens yielded 51 and 44 diploid colonies with robust growth on selective media, respectively (Data S4). Sequencing of prey vectors present in each of these colonies revealed several potential binding partners (Data S4). Of these, an uncharacterized gene predicted to play a role in serine-type endopeptidase inhibitor activity (*Y69H2.3*), genes activated in blocked unfolded protein response (*abu*), and phospholipid scramblases (*scrm*) were found in both libraries with more than two replicates. Given that *abu* genes also commonly appeared in Y2H screens with other *Ophiocordyceps* effector candidates, these interactions were presumably caused by autoactivation and removed from further analysis. We then used BlastP to search the *C. floridanus* proteome (taxid:104421, NCBI) for homologs of the remaining *C. elegans* binding partners. This analysis identified matches for both queries: Y69H2.3 (Camfl2|XM_025412121.1) and SCRM (Camfl2|XM_025407014.1). A closer look at the predicted function revealed that Y69H2.3 homologs had no known function in either worms or ants. In contrast, both of the *C. elegans* SCRM binding partners, SCRM-1 and SCRM-2, aligned with a single ant protein, Phospholipid Scramblase 1 (PS1), which has the same calcium-dependent lipid transporter function. We therefore focused further studies on interactions between *Ophcf2|06345* with SCRM-1 and SCRM-2 (Fig. 2A).

In total, nematodes have eight scramblase proteins. This is a comparatively large number compared to other organisms (e.g., five in *Homo sapiens* and *Mus musculus*, and only two in *Drosophila melanogaster*), while *C. floridanus* ants have just three (PS1, PS3, and PS4). We compared the 3D structures of SCRM-1, SCRM-2, and PS1 to determine if the Ophcf2|06345 peptide would likely bind with them as well given that the amino acid similarity of the *C. elegans* scramblases with PS1 is low (35% for SCRM-1 and 36% with SCRM-2, fig. S2). Comparing AlphaFold2 models with Foldseek^31^ and PyMOL v 2.5.8 (Schrödinger, LLC) showed a much higher degree of similarity (75% and 81% TM scores, respectively), particularly in the core region (fig. S2). This structural similarity was also found between PS1 and the two phospholipid scramblases present in the *D. melanogaster* genome (SCRAMB1, 88%, and SCRAMB2, 76%, TM scores; fig. S2). Additional computational modeling of molecular docking between PS1 and the *Ophcf2|06345* peptide, indicates that the peptide binds to the surface of PS1, presumably acting as a cap to interfere with lipid transport (Fig. 2B). Similar results were obtained with SCRM-1; however, SCRM-2 showed a preference for peptide binding from the side (Fig. 2B). While this sort of binding would be possible with proteins in the nucleoplasm during Y2H analyses, it would not occur *in vivo* due to the transmembrane localization of SCRM proteins, making it a weaker candidate. To verify peptide binding to PS1, and to test whether it binds to any of the other ant scramblase proteins, a series of follow-up pairwise Y2H assays was performed between *Ophcf2|06345* and all three phospholipid scramblase proteins from the *C. floridanus* genome. Results showed that *Ophcf2|06345* only binds to PS1 of *C. floridanus*, consistent with our findings from the *C. elegans* libraries (Fig. 2C).

**Fig. 3.**
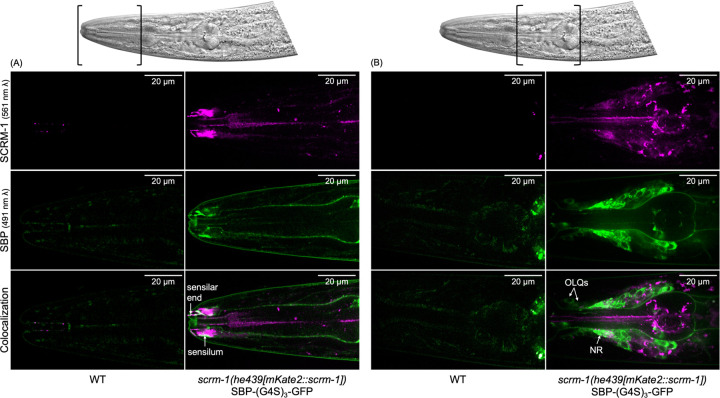
Colocalization of *Ophiocordyceps* Ophcf2|06345 (SBP) and *C. elegans* Scramblase-1. The top images represent spinning disc confocal imagery of the *C. elegans* amphid region, (A), and nerve ring, (B). The top row of fluorescent microscopy images was taken with a 561 nm laser to excite the mKate2 fluorophore linked to SCRM-1, shown in magenta. The middle row of images was taken with a 491 nm laser to excite the GFP fluorophore linked to a codon-optimized variant of SBP, shown in green. The bottom row of images represents a series of composite images with high degrees of colocalization shown in white. The left column in both A and B shows autofluorescence in CGC1 worms while the right columns show fluorescence of the fluorophores in transgenic *scrm-1(he439[mKate2::scrm-1]*) worms expressing pPrab-3_CelOptSBP-(G4S)3-GFP_Tlet-858 (Data S7). Colocalization can be seen to a higher degree in the sensilar tissues, outer labial quadrant neurons (OLQs), and the nerve ring (NR).

Given that SCRM-2 is expressed in very low quantities across all life stages of nematode development (fig. S3), and that knockout studies in *Drosophila* have demonstrated that repair of *SCRAMB1* alone is enough to rescue wild-type phenotypes in Δ*SCRAMB1;*Δ*SCRAMB2* double mutants^24^, we further narrowed the scope of this study to investigate the relationship between the *Ophcf2|06345* peptide and Scramblase-1 homologs, exclusively. We endogenously tagged SCRM-1 with mKate 2 and expressed a GFP-tagged version of *Ophcf2|06345* in *C. elegans* to demonstrated binding *in vivo.* This was confirmed through a high degree of colocalization in the nervous system of *C. elegans* (Fig. 3). Imaging also showed that SCRM-1 is heavily expressed in the sensory organs (Fig. 3A) and the nerve ring (Fig. 3B; Movies S1–2). Taken together, the co-localization data, alongside Y2H and computational evidence, indicate that this previously undescribed Ophcf2|06345 peptide binds to SCRM-1. We therefore named it the “Scramblase-Binding Peptide” (SBP).

### Ophiocordyceps Scramblase-Binding Peptide causes behavioral changes in C. elegans and C. floridanus

**Fig. 4.**
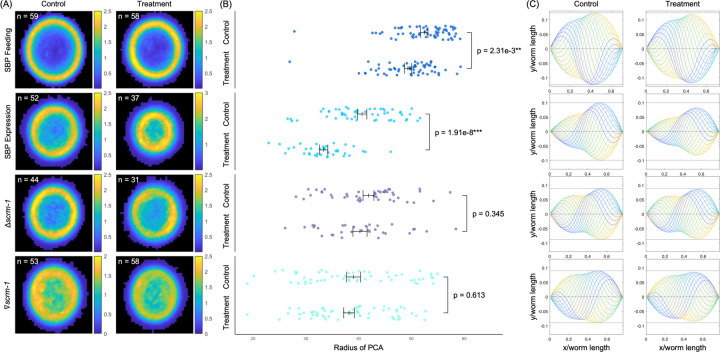
Behavioral analysis of *C. elegans* in response to SBP exposure and SCRM-1 inhibition. The data is organized into rows based on experimental parameters. The top row represents behavioral assays performed with wt nematodes (CGC1) fed SBP-expressing Rosetta^™^ 2 and controls fed empty vector Rosetta^™^ 2. The top middle row represents assays performed with transgenic worms self-expressing SBP and transgenic controls expressing GFP, while the bottom middle row presents behavioral data from *scrm-1*-knockout worms (CU2904) and wt control worms. Finally, the bottom row represents KP3948 worms that were fed HT115(DE3) cells expressing dsRNA for *scrm-1* (Vidal RNAi library; Rual et al., 2004) compared to controls were fed cells producing dsRNA for GFP. (A) Principal component analyses (PCA) of the body curvature of crawling *C. elegans* worms. The joint probability distributions of the amplitudes a_1 (x-axis) and a_2 (y-axis) of the first two modes with the largest variances shows a circular structure. This corresponds to travelling waves along the worm body during motion via lateral undulations. Color denotes the relative fraction of data points in ppm. (B) Average radial distance <\sqrt{a_1^2+a_2^2}> in the space of the first two eigenmodes for each worm. Mean and standard deviation are shown for each data set in black. Significance was determined using a two-tailed t-test. (C) Reconstructed worm shapes from the average contributions of only the first two modes of the PCA (fig. S4) illustrate that the smaller radius corresponds to waves with smaller amplitudes, i.e., smaller peak curvatures.

To determine if the binding of fungal SBP to SCRM-1 can alter behavioral phenotypes, a series of tests was performed with nematodes and analyzed using our new MATLAB software designed for detecting subtle changes in body position^32^. First, we performed two behavioral experiments involving nematodes exposed to SBP. In these tests, the worms were either 1) fed SBP-expressing Rosetta™ 2 bacteria or 2) genetically modified to express codon-optimized SBP. The results were compared to behavioral tests involving the removal or repression of SCRM-1 using the knockout strain CU2904 (Caenorhabditis Genetics Center) and RNAi^33^, respectively. By comparing the results from all four tests, we aimed to link any behavioral changes caused by SBP, at least in part, with the inhibition of SCRM-1 lipid channel transport. In all four conditions, we observed lower body wave amplitudes during nematode crawling (Movie S3), which are depicted by the ring-shaped probability distribution of the first two modes of a principal component analysis in Fig. 4A and fig. S4. However, despite a consistent lower average diameter in all four groups, only the results from the SBP-exposure tests were statistically significant when compared to their respective controls (two-tailed t-test, p-values 2.32e-3** and 1.91e-8***, respectively; Fig. 4B). These behavioral results, in conjunction with the colocalization assays that show an abundance of SCRM-1 in the nerve ring and sensilar organs of *C. elegans*, suggest that SBP can induce behavioral changes presumably through the inhibition of neuromuscular and/or chemosensory systems. Working from this hypothesis, we conducted further behavioral tests in the native ant hosts, *C. floridanus*.

**Fig. 5.**
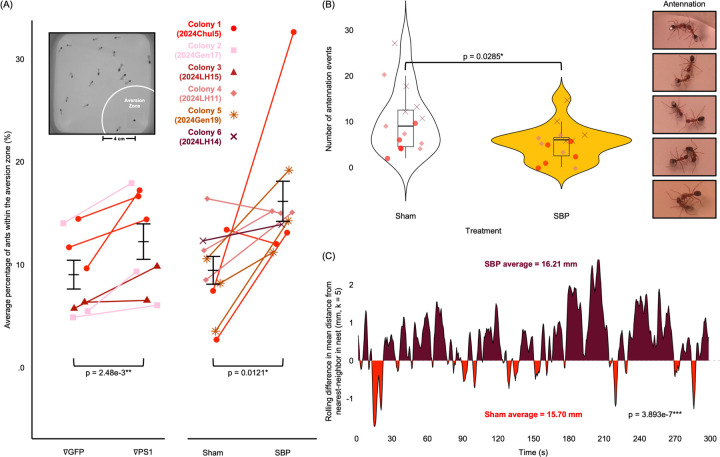
Behavioral analysis of *C. floridanus* in response to SBP exposure and PS-1 repression. (A) The average percentage of ants in the aversion zone (4 cm radius surrounding the citronella pad) calculated as the sum of total time spent by each ant in the zone divided by the total sum time of the recording. Ants injected with dsRNA Phospholipid Scramblase 1 (∇PS1) were compared to those injected with dsRNA for GFP (∇GFP), left. Ants injected with periplasmic protein extracts containing SBP (SBP) produced by Rosetta^™^2 cells were compared to those injected with periplasmic extracts from expressing an empty plasmid (Sham), right. Statistical significance was determined between the means of each group using a one-tailed paired Student’s t-test. (B) The number of antennation events counted between sham-injected ants and between those injected with SBP, examples of which are shown on the right. Statistical significance between the treatment groups was determined using a two-tailed independent Student’s t-test. (C) A difference area plot for the spatio-temporal distribution of ants in a nest environment after injection with either sham treatment or SBP. Numbers on the positive axis represent a higher degree of separation measured in the SBP-injected ants while numbers on the negative axis represent the same for sham-treated individuals. Measurements were taken every 0.5 sec by calculating the nearest-neighbor distance between each ant averaged across the total number of ants in each frame. The values are shown as a rolling average (k = 5) with time on the x-axis. Statistical significance was determined using a linear mixed-effects model.

Similar to the *C. elegans* behavioral assays, *C. floridanus* assays were conducted by 1) injecting ants with SBP extracted from the periplasmic space of the same Rosetta^™^ 2 cells fed to nematodes and 2) repressing PS1 by injecting dsRNA targeting *ps1*. Proper disulfide bond formation for the peptides extracted from the bacterial periplasmic space was verified using a western blot comparing native exacts to those treated with DTT (fig. S5). To record ant behavior, we used a suite of computational tracking software to identify changes in movement and/or chemosensory-related ant behaviors. Compared to controls, both SBP-injected and PS1-repressed ants spent significantly more time near filter pads soaked with citronella oil, a common insect repellant (one-tailed pairwise t-test, p = 2.48e-3** and 0.0121*, respectively; Fig. 5A). These results indicate that the repression of PS1 and exposure to SBP both dampen ant olfactory sensing. This effect was most apparent in the first four minutes of recording, right after the addition of the citronella-soaked pad (fig. S6). Additional pair-wise interaction assays between ants demonstrated that SBP exposure also significantly reduced antennation events compared to controls (two-tailed t-test, p = 0.0285*; Fig. 5B). We also detected a significant reduction in social clustering behavior typically seen between nestmates within the nest, an effect that increased in the SBP-injected group over time (linear mixed-effects model, p < 3.893e-7***; Fig. 5C). Finally, we also observed ants injected with SBP attacking other nestmates during the preparation of behavioral assays (Movie S4), indicating an issue with proper nestmate recognition. Our results, therefore, indicate that *Ophiocordyceps* SBP interferes with host ant chemosensory-related behaviors (e.g., smelling, antennation, and clustering) important for communication and participation in the colony’s social network.

## Discussion

While adaptive manipulation of host behavior is a convergently evolved strategy reported across a wide variety of parasite taxa, the molecular interactions that give rise to these extended phenotypes remain largely unknown. In this study, we identified a novel scramblase-binding peptide (SBP) secreted by *Ophiocordyceps*, which appears to dampen carpenter ant olfactory sensing and lessen communication. Carpenter ants rely heavily on antennal sensilla to identify nest mates, communicate with each other, regulate division of labor, and detect sick individuals^34–35^. This fungal SBP may therefore aid parasitic fitness by preventing nestmate recognition^36^ and thwarting recruitment to the nest via pheromones^37^, ultimately disconnecting the host from its social network. Disrupting smell may also prevent the host itself from recognizing changes in its own cuticular hydrocarbon profile during infection^38^, which could prevent self-grooming behaviors or other seemingly altruistic acts of self-sacrifice and kin selection common in eusocial insects due to their high degree of relatedness^39^. Furthermore, without proper functioning olfactory systems, infected insects might instead rely more heavily on visual systems to guide decision making. This could contribute to light-guided summiting behavior that occurs in later stages of infection^11^. By demonstrating that SBP directly induces host behavioral changes in line with important steps of the fungus’s life cycle, we have successfully characterized the first *Ophiocordyceps* behavioral effector and linked it to its extended phenotype in ants.

In addition to the olfactory effects observed in ants, we were able to identify subtle motor effects using *C. elegans* as a surrogate system. Motor coordination dysregulation is another well-defined step of late-stage behavior manipulation in *C. floridanus* infected by *O. camponoti-floridani*^45^. Towards the end of the infection process, ants exhibit “staggers syndrome”, characterized by a loss of coordination and balance, making the ants appear to “stagger” when walking^46^. However, we were unable to detect any movement effects in our ant studies. This is likely due to the difference in sensitivity of the programs used to screen for these effects in each organism. While our analysis of worm movement was performed using our new MATLAB-based program^32^, our preliminary screening for motor problems in ants coopted existing tools that use simple object tracking (i.e., MARGO^40^). Unlike ants, nematodes move with a travelling body wave^41^, which is measurable with wave function mathematics to characterize precise changes in body positions. This allowed us to detect far more subtle differences in nematode body movement compared to our ant analyses. There is however correlative evidence that suggests subtle motor coordination may be present in ants exposed to SBP as well. The abundance of SBPs localization to neuronal tissues is consistent with findings in *Drosophila* that also found expression of Scramblase 1 and 2 homologs at neuromuscular junctions^24^. Furthermore, Scramblase 1 homologs have also been detected in the brains of mice and are implicated in neurotransmission and synaptic vesicle retrieval of the cerebellum^23^. Given that the cerebellum plays a crucial role in muscle coordination, fine motor movements, and linking sensory detection with movement behaviors^42–44^, it makes sense that we detected motor issues in our more sensitive nematode models. The ability to detect such subtle behavioral effects in *C. elegans* underlines the value of using this model organism in the identification of putative behavioral effectors in complex, non-model systems.

With this study, we have taken the largely descriptive research field of neuroparasitology a step further towards characterizing underlying molecular mechanisms by incorporating model systems. This approach provides a new lab-amenable solution for barriers facing the exploration of novel insecticidal compounds from non-model fungal entomopathogens. Furthermore, our findings suggest that scramblase proteins and their role in neurotransmitter release could provide an alternative target for pre/postsynaptic blockade studied in other fields^19–21^, particularly in response to neuropeptides known to bind to various types of ion-dependent channels of the nervous system and cause pain, muscle paralysis, or even death^20,47–48^. The further exploration of neuropeptides like SBP and their effect on odor-mediated communication in social insects presents a new avenue for the discovery of insecticidal compounds and alternative biocontrol solutions that may spare natural fauna. This work therefore provides a path for screening proteomes for novel effectors and testing their effects on invertebrate nervous systems, particularly those without previously defined function.

## Supplementary Material

Supplement 1

Supplement 2

Supplement 3

Supplement 4

Supplement 5

Supplement 6

Supplement 7

Supplement 8

Supplement 9

Supplement 10

Supplement 11

Supplement 12

Supplement 13

Supplement 14

Supplement 15

Supplement 16

## Data Availability

Files for 3D printing the replica plating apparatus used in this study can be found at https://github.com/WCBeckerson/3D-Print-Replica-Plating-Apparatus. All 512 nematode and 88 ant video files analyzed in this study are deposited at Zenodo under the DOIs 10.5281/zenodo.16760960 and 10.5281/zenodo.16760416, respectively.
